# Authors’ Reply to: Clarity on the Type of Review. Comment on “Value Cocreation in Health Care: Systematic Review”

**DOI:** 10.2196/39397

**Published:** 2022-07-11

**Authors:** Yuxin Peng, Tailai Wu, Zhuo Chen, Zhaohua Deng

**Affiliations:** 1 School of Medicine and Health Management Tongji Medical College Huazhong University of Science and Technology Wuhan China; 2 Department of Health Policy and Management College of Public Health University of Georgia Athens, GA United States; 3 School of Economics Faculty of Humanities and Social Sciences University of Nottingham Ningbo China Ningbo China; 4 School of Management Huazhong University of Science and Technology Wuhan China

**Keywords:** value cocreation, health care, patient value, health care professional value, systematic review

We thank Kajal [1] and the editors of the *Journal of Medical Internet Research* for providing this opportunity to discuss our paper [2] with an academic audience directly after the publication of our work.

Overall, we think our systematic review is not perfect, but we endeavor to bring contributions and values to health care knowledge. We believe our audience can find not only the flaws but also the values of our paper. We, along with the reviewers and editors of the *Journal of Medical Internet Research*, have worked together to make this systematic review as valuable as possible during the publication process; we hope the readers will benefit from it in their future studies.

Our specific responses to Kajal [1] are as follows: First, we believe our study is a systematic review rather than a scoping review since our review not only identified available studies but also identified principal results and areas for future research [3]. The integrative framework provided in our review could serve as the basis for decision-making in value cocreation in health care. We understand that scoping reviews and systematic reviews overlap with each other, but our review matches the methods of a systematic review. Moreover, if the audience read our paper more carefully, they will find that “this area of research is new, and literature is fragmented” is not our only motivation; we also propose other motivations, including “for VCCH, the factors are not explored systematically, underlying mechanisms of its factors are vague, and consequences are not fully investigated” [2]. Finally, we may not have formally proposed a research question in our review, but we did have a specific research aim with the following implied question: What are the dimensions, antecedents, and consequences of value cocreation in health care, and how do they relate?

Second, we think our current search terms are adequate for our review goals. We have tried other search terms related to our research topic, but not many related or qualified articles were found.

Third, the risk of biases and heterogeneity were assessed using the MMAT (Mixed Methods Appraisal Tool). This tool not only appraises the quality of individual studies given the heterogeneity of the study designs but also accounts for many biases including confounding bias, nonresponse bias, and sampling bias [4]. Meanwhile, many previous systematic reviews or systematic review protocols have used the MMAT to assess the risk of bias, such as Xu et al [5], Pearson et al [6], and Gledhill et al [7].

Forth, we admit that developing and presenting a theoretical framework is not a standard method, but it is our unique way of contributing to knowledge in health care. As you can see in our paper, the framework could (1) map and visualize studies systematically, (2) provide a novel theoretical perspective, (3) and imply many future research directions directly. Regarding these 3 benefits, we believe it is necessary to present this framework even though it is not a standard method.

We hope our response has alleviated the concerns raised by Kajal [1].

## Abbreviations

MMAT: Mixed Method Appraisal Tool

## References

[1] Kajal F (2022). Clarity on the Type of Review. Comment on "Value Cocreation in Health Care: Systematic Review". J Med Internet Res.

[2] Peng Y, Wu T, Chen Z, Deng Z (2022). Value cocreation in health care: Systematic review. J Med Internet Res.

[3] Munn Z, Peters MDJ, Stern C, Tufanaru C, McArthur A, Aromataris E (2018). Systematic review or scoping review? Guidance for authors when choosing between a systematic or scoping review approach. BMC Med Res Methodol.

[4] Hong QN, Fàbregues S, Bartlett G, Boardman F, Cargo M, Dagenais P, Gagnon M, Griffiths F, Nicolau B, O’Cathain A, Rousseau M, Vedel I, Pluye P (2018). The Mixed Methods Appraisal Tool (MMAT) version 2018 for information professionals and researchers. EFI.

[5] Xu N, Lv A, Li T, Li X, Huang M, Su Y (2021). Experiences of healthcare providers during the coronavirus pandemic and its impact on them: protocol for a mixed-methods systematic review. BMJ Open.

[6] Pearson Sally Anne, Taylor Sally, Marsden Antonia, Yorke Janelle (2021). Access to systemic anti-cancer therapies for women with secondary breast cancer-protocol for a mixed methods systematic review. Syst Rev.

[7] Gledhill Adam, Forsdyke Dale, Murray Eliot (2018). Psychological interventions used to reduce sports injuries: a systematic review of real-world effectiveness. Br J Sports Med.

